# In memory of tachycardia: A wide complex tachycardia in a young male

**DOI:** 10.1002/joa3.12385

**Published:** 2020-06-18

**Authors:** Gregory P. Siroky, Seth Keller, Ranjit Suri

**Affiliations:** ^1^ Department of Cardiology Division of Electrophysiology Mount Sinai Morningside Icahn School of Medicine at Mount Sinai New York NY USA

**Keywords:** cardiac memory, catheter ablation, fascicular ventricular tachycardia

## Abstract

We present a case of a 24‐year‐old male with palpitations and a wide complex tachycardia. Baseline electrocardiogram (ECG) after termination of tachycardia demonstrates a normal rhythm but with inferior/anterolateral T‐wave inversions (TWIs). Electrophysiologic study confirmed the diagnosis of posterior fascicular ventricular tachycardia successfully terminated by anatomic ablation of the left posterior fascicle. TWIs on the patient's baseline ECG were consistent with cardiac memory.

## Graphical abstract

Twenty‐four‐year‐old male presents with palpitations and wide complex tachycardia on ECG. Baseline ECG after termination of tachycardia demonstrates a normal rhythm but with inferior/anterolateral T‐wave inversions (TWIs). Electrophysiologic study confirms the diagnosis of posterior fascicular ventricular tachycardia with successful anatomic ablation of the left posterior fascicle. TWIs were consistent with cardiac memory.

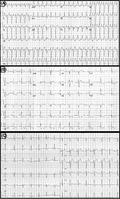

## 1 CASE

**FIGURE 1:**
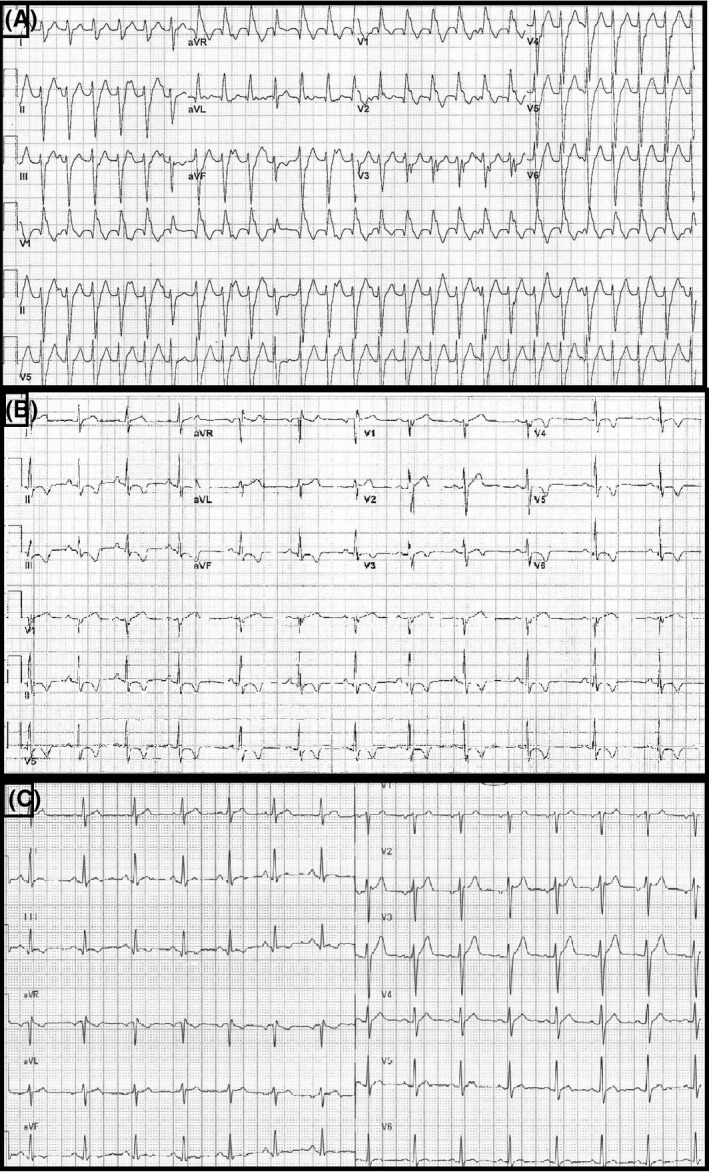
A, Initial 12‐lead ECG obtained at urgent care center. B, Repeat 12‐lead ECG after cessation of tachycardia. C, 12‐lead ECG obtained a short while after intervention to treat arrhythmia as seen in (A)

A 24‐year‐old male with no past medical history presents to an urgent care center with palpitations. Initial ECG obtained is shown in Figure 1A. Transthoracic echocardiogram (TTE) obtained in the office demonstrated global left ventricular (LV) dysfunction with an ejection fraction (EF) of 35%. In the hospital, administration of intravenous amiodarone terminated the patient's tachycardia and a subsequent ECG was obtained (Figure 1B). Repeat TTE showed normalization of the EF to 55% and cardiac magnetic resonance imaging demonstrated a normal LV without evidence of myocardial pathology. What is the most likely diagnosis of the tachycardia? What is the etiology of the ECG abnormalities seen in Figure 1A and what intervention was performed leading to the ECG in Figure 1C?

## 2 DISCUSSION

**FIGURE 2:**
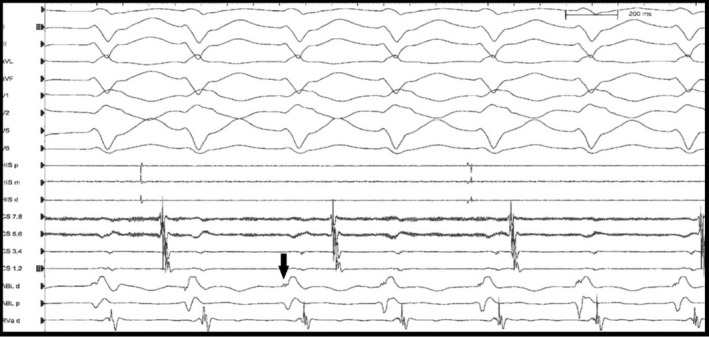
Surface and intracardiac electrograms during induced posterior fascicular ventricular tachycardia. Black arrow points to purkinje potential of left posterior fascicle on distal ablation catheter which was targeted for ablation. Top to bottom—bipolar leads I, II, and III; augmented limb leads augmented vector left (aVL) and aVF; precordial leads V1, V2, V5, and V6; HIS catheter proximal (p), mid (m), and distal (d); coronary sinus (CS) catheter proximal bipoles (7,8) to distal bipoles (1,2); ablation catheter (ABL) d and p; RVad, right ventricular apex distal

The initial ECG shows a wide complex tachycardia (WCT) at 160 bpm, left‐axis deviation of −35, QRS duration of 146 ms, and morphology demonstrating a right bundle branch (RBBB) and left anterior fascicular block (LAFB) pattern. The differential diagnosis for such a tachycardia includes a supraventricular tachycardia (SVT) with RBBB/LAFB aberrancy, an SVT with bystander nodofascicular (NF) accessory pathway (AP) connecting the slow AV nodal pathway to the left posterior fascicle,^1^ an orthodromic reentrant tachycardia using a concealed NF AP^2^, or a left posterior fascicular (LPF) ventricular tachycardia (VT) using the LPF as the retrograde limb of the circuit. Inspection of the ECG shows atrioventricular dissociation (clearly seen in lead II of the rhythm strip) and fusion complexes (6th and 10th QRS complexes) making the diagnosis of LPF VT more of a certainty. During electrophysiology study, the clinical tachycardia was induced (Figure 2) after infusion of isoproterenol but would terminate during attempts at entrainment mapping. Therefore, anatomic‐based ablation^3^ of the distal one third of the LPF was performed (Figure S1), rendering the tachycardia noninducible. An additional endpoint of ablation was the appearance of new q‐waves in the inferior limb leads (II, III, and augmented vector floor (aVF)) as a result of ablating the distal third of the LPF with initial electrical forces directed superiorly (Figure S2).

Now turning attention to the abnormalities seen in the baseline ECG, specifically the inferior/anterolateral T‐wave inversions (TWI). There are numerous causes of TWI, most which are of clinical concern, however, there are a few etiologies which are less sinister and quite benign. A clinician seeing these abnormalities in conjunction with a diagnosis of VT would be concerned with myocardial ischemia thereby triggering a cascade of events leading to coronary artery evaluation and exposing the patient to unnecessary testing and potential risks. Taking into consideration the patient's age, palpitations, and lack of chest pain, myocardial ischemia is less likely. In light of the diagnosis of VT, the more reasonable cause of the TWI is cardiac memory or T‐wave memory (TWM). Cardiac memory results from abnormal ventricular activation leading to short‐ and long‐term alterations on the molecular level by which repolarization changes are achieved and can occur after ventricular pacing, after resolution of left bundle branch block, after ablation of an AP, and posttermination of VT.^4^, ^5^ It can sometimes be challenging to differentiate TWI as a result of TWM vs myocardial ischemia, however, Shvilkin et al proposed two criteria in favor of TWM: (a) positive T‐waves in leads I and aVL; and (b) precordial TWI deeper than inferior TWI, both of which fit our patient's ECG.^5^ ECG obtained 1 week after ablation demonstrated resolution of the TWI (Figure 1C) owing to the success of the ablation without further episodes of palpitations and confirming the diagnosis of TWM.

In conclusion, this case highlights two important concepts: (a) the correct diagnosis of a WCT is imperative to a patient's management; and (b) TWI can be a temporary, physiologic remnant of abnormal ventricular activation as opposed to myocardial ischemia.

## DISCLOSURES

None.

## Supporting information

Fig S1

Fig S2

## References

[1] Hoffmayer KS , Lee BK , Vedantham V , Bhimani AA , Cakulev IT , Mackall JA , et al. Variable clinical features and ablation of manifest nodofascicular/ventricular pathways. Circ Arrhythm Electrophysiol. 2015;8:117–27. 25472957 10.1161/CIRCEP.114.001924

[2] Sternick E , Wellens HJ . Variants of ventricular preexcitation. Malden, MA: Blackwell Publishing; 2006 p. 79.

[3] Lin D , Hsia HH , Gerstenfeld EP , Dixit S , Callans DJ , Nayak H , et al. Idiopathic fascicular left ventricular tachycardia: linear ablation lesion strategy for noninducible or nonsustained tachycardia. Heart Rhythm. 2005;2:934–9. 16171747 10.1016/j.hrthm.2005.06.009

[4] Wellens HJ . QRS complex change during carotid sinus massage in ventricular pre‐excitation. Heart Rhythm. 2019;16(8):1296–8. 31358267 10.1016/j.hrthm.2019.03.020

[5] Shvilkin A , Huang H , Josephson ME . Cardiac memory: diagnostic tool in the making. Circ Arrhythm Electrophysiol. 2015;8(2):475–82. 25900990 10.1161/CIRCEP.115.002778

