# Supplementary material for: In memory of tachycardia: A wide complex tachycardia in a young male
Source: J Arrhythm. 2020 Jun 18;36(4):801–3. doi: 10.1002/joa3.12385 (PMC7411235; doi:10.1002/joa3.12385)
Supplement: Supplementary file 1 — Fig S1 [file JOA3-36-801-s001.pdf]

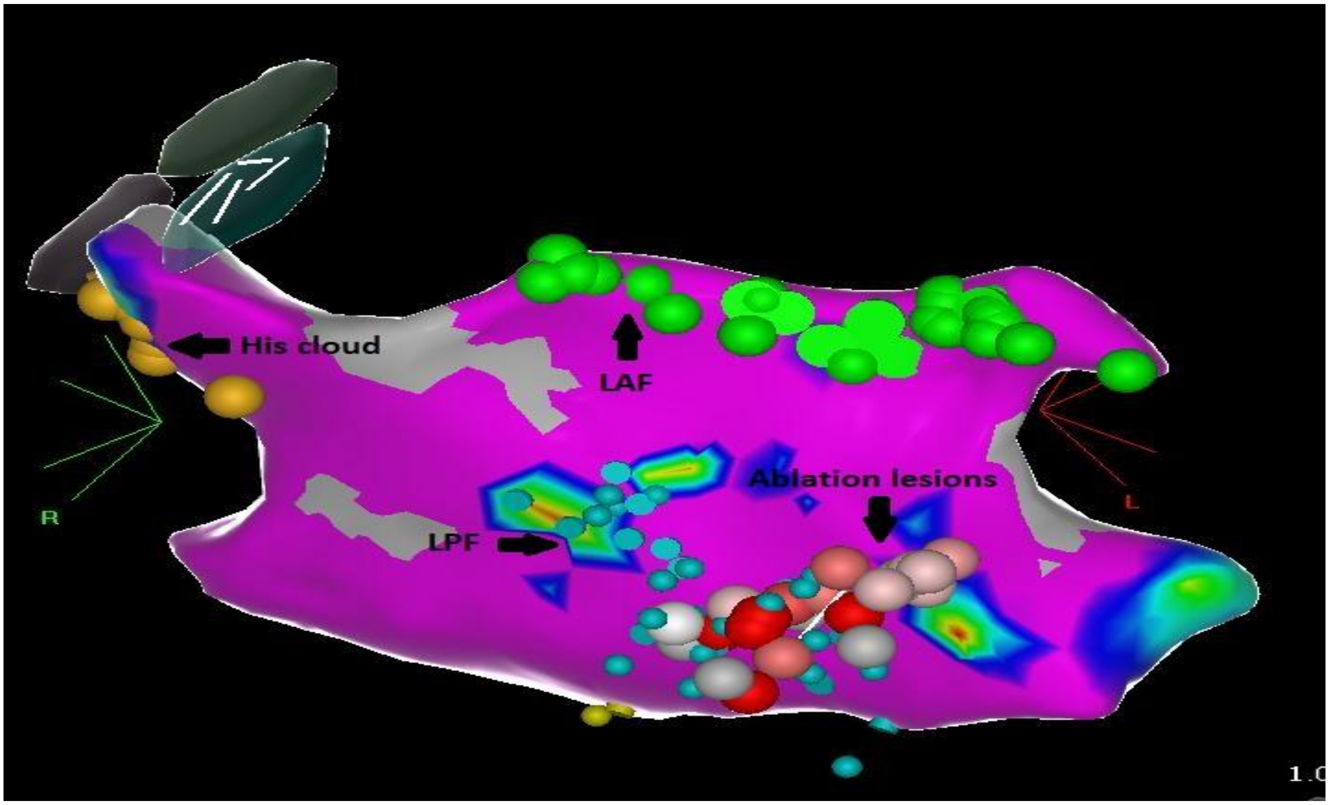

Supplementary Figure 1. Electroanatomic map of left ventricular septum as viewed from the right anterior oblique projection (RAO). Areas of the His bundle location/cloud (yellow dots), left anterior fascicle (LAF – green dots), and left posterior fascicle (LPF – blue dots) were marked prior to ablation of the distal third of the LPF (red/pink dots).
