# Supplementary material for: In memory of tachycardia: A wide complex tachycardia in a young male
Source: J Arrhythm. 2020 Jun 18;36(4):801–3. doi: 10.1002/joa3.12385 (PMC7411235; doi:10.1002/joa3.12385)
Supplement: Supplementary file 2 — Fig S2 [file JOA3-36-801-s002.pdf]

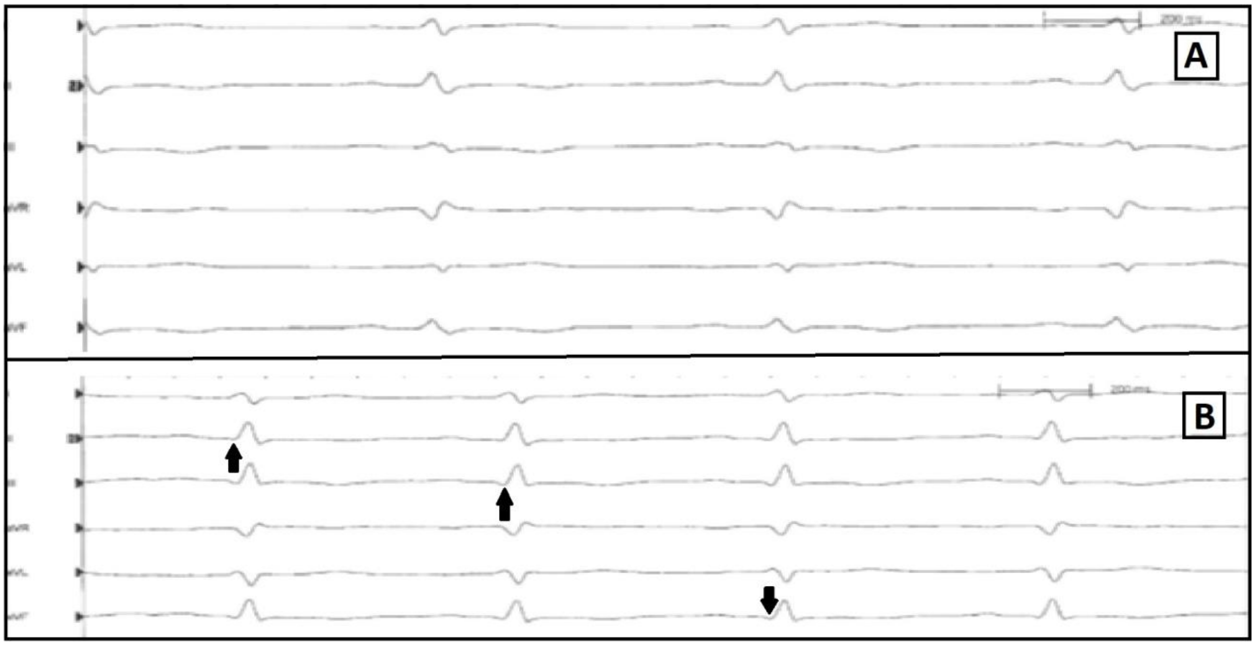

Supplementary Figure 2. Pre- (A) and post-ablation (B) ECGs demonstrating new q-waves in the inferior limb leads (II, III, and aVF) representing ablation of the distal portion of the left posterior fascicle with initial forces directed superiorly.
